# The association between the quality and quantity of carbohydrate intake and the size, depth, and Wagner grade of diabetic foot ulcers in patients with type 2 diabetes

**DOI:** 10.1371/journal.pone.0323537

**Published:** 2025-06-11

**Authors:** Faezeh Geravand, Ensieh Nasli-Esfahani, Mohsen Montazer, Moharam Jalalzadeh, Leila Azadbakht

**Affiliations:** 1 Department of Community Nutrition, School of Nutritional Sciences and Dietetics, Tehran University of Medical Sciences, Tehran, Iran; 2 Diabetes Research Center, Endocrinology and Metabolism Clinical Sciences Institute, Tehran University of Medical Sciences, Tehran, Iran; 3 Department of Community Nutrition, School of Nutrition and Food Science, Isfahan University of Medical Sciences, Isfahan, Iran; University of Cape Town, SOUTH AFRICA

## Abstract

**Aims:**

This cross-sectional study aimed to investigate the association between carbohydrate quantity and quality and the size, depth, and Wagner grade of diabetic foot ulcers (DFUs).

**Methods:**

The study was conducted on 300 participants with DFUs at the Diabetes Clinic of Tehran University of Medical Sciences. Dietary intake was assessed using three 24-hour dietary recalls. Anthropometric measurements, physical activity levels, and socioeconomic factors were evaluated. The location, Wagner grade, length, width, and depth of the diabetic foot ulcer, were assessed by reviewing the patient’s medical records and utilizing the information recorded therein.

**Results:**

The findings indicated that out of all the carbohydrate indices examined, only the ratio of whole grain to total grain intake had a significant association with the length of the diabetic foot ulcer. Specifically, participants who consumed lower amounts of whole grain had larger diabetic foot ulcers compared to those with higher whole grain intake (0.85 Vs 0.54, P_value _= 0.04). This relationship remained significant even after adjusting for potential confounding factors such as age, gender, total energy intake, smoking, physical activity, good shoe condition, and BMI (0.71 Vs 0.52, P_value_ = 0.03).

**Conclusion:**

The findings emphasize the significant role of whole grain intake in the healing process of DFUs. However, further research with larger sample sizes is needed to investigate the impact of other carbohydrate indices.

## Introduction

Diabetic foot ulcers (DFUs) are a significant complication that arises due to neurological abnormalities and varying degrees of peripheral vascular abnormalities in the lower limbs [1,2]. A substantial number of diabetic patients, ranging from 9.1 to 26.1 million, develop diabetic foot ulcers annually [3]. Diabetic foot ulcers and their complications have a significant negative impact. They diminish both physical and mental quality of life. These ulcers also increase the risk of infections, recurrent ulcers, lower limb amputations, and even higher mortality rates. Moreover, these complications impose substantial financial burdens due to the need for frequent and prolonged hospitalizations [4–11].

Wound healing is influenced by several factors, including wound site infection, poor glycemic control, and developing nephropathy, also vasculopathy. The nutritional status of patients plays a critical role in the wound healing process [12–15]. Alongside these factors, insulin resistance is recognized as an important contributor to the pathogenesis and healing of ulcers [16], The type and amount of carbohydrates in someone’s diet can impact their glycemic control and insulin resistance. These factors play a crucial role in the context of wound healing [17,18]. Dietary fiber, has been recognized for its potential benefits in glycemic control. Studies have shown that higher dietary fiber intake is associated with improved glycemic control, and lower risk of developing diabetes complications such as insulin resistance [19,20]. Besides, the ratio of whole grains to total grains consumed has been suggested as an important measure of the quality of carbohydrate intake. A higher ratio indicates a healthier dietary pattern [21]. Moreover, glycemic load, a measure that takes into account both the quality and quantity of dietary carbohydrates, has been implicated in glycemic control and the development of diabetes complications. Diets with a high glycemic load, which cause rapid increases in blood glucose levels after meals, have been linked to poorer glycemic control, insulin resistance, and an elevated risk of other diabetes-related complications [22]. Despite the recognized impact of dietary factors on wound healing, the specific relationship between dietary composition, such as glycemic load, and characteristics of diabetic foot ulcers (DFUs) - including size, depth, and Wagner grade - has not been extensively studied. Understanding these associations is crucial for developing targeted dietary interventions and optimizing management strategies for individuals with DFUs. Therefore, the present study aims to investigate the association of dietary composition, particularly carbohydrate and fiber ratios, with the size, depth, and Wagner grade of DFUs in patients with type 2 diabetes. By exploring these associations, we aim to shed light on the potential influence of dietary factors on DFU characteristics. Furthermore, finding significant relationships between dietary composition, glycemic load, and characteristics of diabetic foot ulcers (DFUs) could help develop evidence-based dietary recommendations specifically tailored for individuals with DFUs or at risk of developing them.

## Materials and methods

### Study design and participants

The present cross-sectional study was conducted from 2022 until 2023 on patients with diabetic foot ulcers at the Diabetes Research Center affiliated by the Research Institute of Endocrinology and Metabolism, which is part of Tehran University of Medical Sciences. The study was conducted in accordance with the ethical guidelines of the Nutrition Faculty Ethics Committee at Tehran University of Medical Sciences, following the Helsinki Declaration (identification number: IR.TUMS.MEDICINE.REC.1401.544). Written informed consent was obtained from all participants. Accounting for a 5% type I error rate and 80% power, and considering the size and depth of the diabetic foot ulcers as the key variables, the required sample size for this cross-sectional study was calculated to be 300 individuals using the proposed formula [23–28]:


$$m^{\prime }=\frac{\;[ca/2\sqrt{{(r}_{+1}\overset{―}{)p}\overset{―}{Q}}-C_{1-B}{\sqrt{r_{1}P_{1}Q_{1}+p_{2}Q_{2}}]}^{2}}{r(p2-p1)2}$$

$$\text{m}=\frac{\text{ḿ}}{4}{\left(1+\sqrt{1+\frac{2\left(\text{r}+1\right)}{ḿr|P_{2}-P_{1}|}}\right)}^{2}$$

Convenience sampling was employed. In this study, individuals who met the following criteria were included: having type 2 diabetes for at least 2 years documented in the patient’s medical record, aged between 25 and 65 years, and the presence of a diabetic foot ulcer. Individuals with the following conditions were excluded from the study: having incomplete medical records, being pregnant or lactating, and adherence to a specific dietary regimen. Regarding to rechecking, those with reported energy intake of <800 or >4200 kcal/d were excluded. All study participants provided written consent after being fully informed about the study. Informed consent for all illiterate participants was obtained from their legal guardians.

### Anthropometric assessment

Weight was measured using a digital scale with an accuracy of 100 grams from Seca, Hamburg, Germany, while participants wearing minimal clothing and no shoes. Height was measured following a standard protocol using a wall stadiometer with an accuracy of 0.1 cm. The body mass index (BMI) was then calculated by dividing the weight in kilograms (kg) by the square of the height in meters (m^2^).

### Physical activity

It was evaluated using the International Physical Activity Questionnaire-short form (IPAQ-SF) [29]. The assessment involved determining the level of physical activity, with metabolic equivalent-hours per day (MET-h per day) used as the unit of measurement for expressing this value.

### Socioeconomic status

Data on socioeconomic status was obtained using a Persian socioeconomic questionnaire that had been validated. The questionnaire included various indicators such as income, residential area, family size, educational level, ownership of vehicles (including type and quantity, if applicable), real estate ownership or tenancy, and the number of bedrooms [30,31].

### Assessment of dietary intake

Dietary intake assessment in the present study was conducted using three 24-hour dietary recalls. To gather nutritional data from the study participants, researchers conducted face-to-face interviews. In these interviews, individuals were asked to report their food and beverage consumption from the previous day, including details such as the type of items, ingredients, timing of meals, and number of meals. The 24-hour dietary recalls obtained were reviewed immediately by the researcher to address any potential discrepancies or clarifications with the study participants. Twenty-four-hour dietary recalls were collected from two non-consecutive weekdays and one weekend day. All reported food items were converted to grams per day and entered into the Nutritionist IV software (N-Squared Computing, Version 4.0, Cincinnati, OH, USA), where energy and nutrient intakes were calculated for each individual [32].

### Assessment of the quality and quantity of carbohydrates

The carbohydrate content of the patients’ diet was calculated as a percentage of daily calorie intake and grams per day. The Carbohydrate Quality Index (CQI) was used as an indicator of carbohydrate quality in the diet. It includes the total dietary fiber intake (grams per day), glycemic load, ratio of whole grain carbohydrates to total grain carbohydrates, and the ratio of carbohydrates in solid foods to total carbohydrates (solid carbohydrates + liquid carbohydrates) [21,33]. Liquid carbohydrates include sweetened beverages and fruit juices, while the carbohydrate content of the remaining carbohydrate-based food items was considered to be in the form of solid carbohydrates.[34]. The values related to the glycemic index were extracted from the book “Glycemic Index Indicators” written by Dr. Talaban, the glycemic index table of foods from Australia, and a published article in the American Journal of Clinical Nutrition. For foods that were not listed in the tables, the average of similar foods that were available was calculated. For each individual, the GI values from food items were multiplied by the carbohydrate intake (in grams) from that food item, and then summed to obtain the GL. By including the glycemic index value, carbohydrate, and fiber content of each consumed food item over the course of a day, the daily glycemic index of the dietary intake was obtained [21,35–43].

Σ (GI × available carbohydrate)/total available carbohydrate)
(available carbohydrate = total carbohydrate – total fiber)
The glycemic load (GL) of the dietary intake was also calculated using the following method:
(total GI ×total available carbohydrate)/ 100

### Assessment of diabetic foot ulcers

The trained physician and nurse reported the information related to the foot ulcers based on the IWGDF (International Working Group on the Diabetic Foot) guidelines in the patients’ medical records [44]. Then, we extracted the information from the patients’ medical records. Information about the patients’ HbA1c was extracted from the reports recorded in the patients’ medical records. The Wagner-Meggitt system was used to classify the severity of foot wounds [45]. The location, infection, length, width, and depth of the diabetic foot ulcer, were assessed by reviewing the patient’s medical records and utilizing the information recorded therein. The presence of neuropathy was evaluated in both feet using a 10-gram monofilament test at 10 specific sites [46,47]. Patients were then classified into three categories based on their sensitivity to the monofilament: those with normal sensation at more than 8 sites, those with reduced sensation at 4–7 sites, and those with absent sensation at fewer than 3 sites [48,49]. Peripheral arterial disease (PAD) was detected through the use of the ankle-brachial index (ABI). The ABI was calculated as the ratio of the highest systolic blood pressure measured at the ankle to the highest systolic blood pressure measured at the brachial artery [50,51]. Participants were categorized into two groups based on their ABI values: those with an ABI < 0.90 were classified as the “Low ABI Group,” indicating the presence of PAD, while those with an ABI ≥ 0.90 were classified as the “Normal ABI Group,” indicating normal arterial function. In addition, those with an ABI value of 1.3 or higher, indicated a noncompressible calcified vessel [50,52,53]. All these measurements and examinations were performed by the physician and nurse based on IWGDF.

### Statistical analysis

We divided the participants into categories based on tertiles of their dietary fiber and glycemic load (GL) values. For continuous variables, such as the length, width, and depth of diabetic foot ulcer, we used analysis of variance (ANOVA) to compare general characteristics. For categorical variables, we employed Pearson’s chi-square test. ANOVA was utilized to determine the means of length, width, and depth of diabetic foot ulcer across tertiles of dietary fiber and GL. Additionally, we conducted an analysis of covariance (ANCOVA) to assess the significance of foot ulcer indices across tertiles of carbohydrate intake, while taking into account covariates such as age, sex, and daily energy intake. Logistic regression was utilized to calculate the multivariable adjusted odds ratio for foot ulcer indices across tertiles of carbohydrate intake. Statistical analyses were conducted using SPSS (Statistical Package for the Social Sciences), version 26, developed by SPSS, Inc. in Chicago, IL. A significance level of p < 0.05 was used to determine statistical significance.

## Results

General characteristics are indicated across the tertiles of carbohydrate intake “Table 1”. The lowest tertile corresponds to the least amount, while the highest tertile represents the highest intake. Each tertile of carbohydrate intake consists of 100 participants. The mean age of participants was significantly higher in T1 compared to T3 (62.1 vs 57.5, P = 0.005). Additionally, a higher proportion of men had foot ulcers in the third tertile compared to the first tertile (P < 0.001). Furthermore, in the third tertile, significantly more patients were smokers compared to the first tertile. However, all other demographic characteristics did not show significant differences between T1 and T3. However, there was one exception. In relation to the absence of monofilament sensation, a higher percentage of individuals with greater carbohydrate intake exhibited neuropathy, which was statistically borderline significant (P = 0.058).

**Table 1:** The overall characteristics of individuals with type 2 diabetes and diabetic foot ulcers across tertiles of carbohydrate intake.

	Tertiles of carbohydrate intake			
	T1 (n = 100) <225.7	T2 (n = 100) 225.7-302.3	T3 (n = 100) >302.3	P^a^
Age(years)	62.11 ± 10.72	60.81 ± 9.53	57.52 ± 10.51	0.005
BMI(kg/m2)	29.34 ± 4.94	27.97 ± 3.95	29.28 ± 4.87	0.062
Physically active(%) (High)	1.01	0.72	3.03	0.147
Men (%)	15.32	23.07	25.09	<0.001
Married (%)	26.81	29.13	26.80	0.528
SES(%) (High)	2.37	2.35	4.02	0.093
Smoking(%)	3.73	8.06	9.04	0.012
Family member(%) (low)	29.25	28.96	28.91	0.606
Covid-19 history (%)	17.06	16.73	16.00	0.911
Suitable shoe condition (%)	7.86	9.82	7.86	0.423
Foot ulcer history (%)	15.50	18.97	17.22	0.322
Insulin (%)	21.35	18.77	20.75	0.484
Foot ulcer infection (%)	4.73	7.32	5.76	0.390
ABI	1.13 ± 0.18	1.16 ± 0.17	1.17 ± 0.17	0.419
Sensitivity to monofilament-absent	20.90	21.50	24.8	0.058
HbA1c (%)	8.89 ± 2.05	10.06 ± 10.33	12.07 ± 66.76	0.344

Abbreviations: BMI, body mass index; SES, socioeconomic status.

All values are means ± standard deviation (SD) unless indicated.

^a^Obtained from ANOVA for continuous variables and chi-square test for categorical variables.

Dietary intakes are outlined in “Table 2”. Patients who consumed higher amounts of carbohydrates also had higher energy intake, significantly more fiber, and greater consumption of vitamins B1, C, E, and phosphorus compared to those with the lowest carbohydrate intake.

**Table 2:** Multivariable-adjusted intakes of nutrients among participants with type 2 diabetes and diabetic foot ulcers across tertiles of carbohydrate intake.

	Tertiles of carbohydrate intake			
	T1 (n = 100)	T2 (n = 100)	T3 (n = 100)	P^a^
Daily energy intake(kcal/d)	1553.9 ± 375.7	2130.1 ± 330.4	2780.4 ± 601.7	<0.001
VitaminA (RAE/d)	810.5 ± 558.2	1064.9 ± 775.2	1145.8 ± 1311.8	0.309
Vitamin D (mcg/d)	0.4 ± 0.9	0.6 ± 1.2	0.6 ± 1.0	0.410
Vitamin E (mg/d)	2.2 ± 1.2	3.7 ± 3.1	3.5 ± 1.8	0.005
Vitamin K (mg/d)	91.1 ± 76.4	120.8 ± 92.2	131.3 ± 92.2	0.134
Vitamin B1 (mg/d)	1.3 ± 0.3	1.8 ± 0.2	2.5 ± 0.6	<0.001
Vitamin B2 (mg/d)	1.2 ± 0.4	1.5 ± 0.4	2.0 ± 0.9	0.072
Vitamin B3 (mg/d)	15.3 ± 5.3	20.4 ± 4.3	27.1 ± 7.9	0.656
Vitamin B6 (mg/d)	1.1 ± 0.5	1.5 ± 0.7	1.9 ± 0.9	0.699
Vitamin B9 (mcg/d)	168.4 ± 72.4	214.0 ± 78.3	248.1 ± 106.3	0.256
Vitamin B12 (mcg/d)	3.5 ± 3.9	3.5 ± 2.9	5.7 ± 13.9	0.303
Vitamin C (mg/d)	88.6 ± 53.9	122.8 ± 74.6	150.8 ± 108.2	0.008
Calcium (mg/d)	568.8 ± 231.6	767.3 ± 293.0	991.4 ± 409.8	0.977
Phosphorus (mg/d)	802.8 ± 282.5	998.0 ± 311.4	1176.3 ± 420.2	0.037
Iron (mg/d)	11.2 ± 3.1	15.2 ± 3.2	20.5 ± 8.0	0.733
Dietary fiber (g/d)	12.9 ± 5.5	17.6 ± 5.1	21.3 ± 7.7	0.001

All values are means ± standard error (SE); all values are adjusted for energy intake.

^a^Obtained from ANCOVA.

The foot ulcer indices did not show any statistically significant differences among the tertiles of carbohydrate intake and whole grain/total grain, except for one measure “Tables 3 and 4”. The mean ulcer width was slightly lower in individuals who had a higher ratio of whole grains to total grains. This difference was statistically marginally significant (P = 0.06). As the ratio of carbohydrate from solid food to total carbohydrate increased, the length of the diabetic foot ulcer also increased, but it was not statistically significant “Table 4”. Participants with a greater size of diabetic foot ulcer had lower fiber intake but higher glycemic load, however it was not statistically substantial “Table 5”.

**Table 3:** Diabetic Foot ulcer indices based on amount of carbohydrate tertiles in type 2 diabetic patients.

	Tetiles of carbohydrate intake			
	T1 (n = 100)	T2 (n = 100)	T3 (n = 100)	P^a^
Foot ulcer length	1.40 ± 0.17	1.15 ± 0.13	1.18 ± 0.17	0.51
Foot ulcer width	0.85 ± 0.09	0.64 ± 0.07	0.79 ± 0.09	0.14
Foot ulcer depth	0.36 ± 0.07	0.26 ± 0.05	0.22 ± 0.07	0.49

All values are means ± standard error (SE); all values are adjusted for age, gender and energy intake, smoking, socioeconomic status, physical activity, insulin intake, more than 2years of diabetes, debridement, good shoe condition, good socks condition, diabetic foot ulcer history, body mass index.

^a^Obtained from ANCOVA.

**Table 4:** Diabetic foot ulcer indices based on whole grain to total grain and carbohydrate of solid food to total carbohydrate tertiles in type 2 diabetic patients.

	whole grain/total grain				Carbohydrate of solid food/ total carbohydrate			
	T1(n = 101) < 0.14	T2(n = 98) 0.14–0.40	T3(n = 101) > 0.40	P^2^	T1(n = 110) < 0.86	T2(n = 89) 0.86–0.91	T3(n = 101) > 0.91	P^a^
Foot ulcer length	1.37 ± 0.13	1.27 ± 0.13	1.08 ± 0.13	0.29	1.15 ± 0.12	1.24 ± 0.14	1.33 ± 0.13	0.59
Foot ulcer width	0.89 ± 0.07	0.71 ± 0.07	0.66 ± 0.07	0.06	0.75 ± 0.06	0.78 ± 0.07	0.75 ± 0.07	0.95
Foot ulcer depth	0.25 ± 0.05	0.31 ± 0.05	0.28 ± 0.05	0.71	0.30 ± 0.05	0.34 ± 0.06	0.21 ± 0.05	0.30

All values are means ± standard error (SE); all values are adjusted for age, gender and energy intake, smoking, socioeconomic status, physical activity, insulin intake, more than 2years of diabetes, debridement, good shoe condition, good socks condition, foot ulcer history, body mass index.

^a^Obtained from ANCOVA.

**Table 5:** Diabetic foot ulcer indices based on glycemic load and dietary fiber tertiles in type 2 diabetic patients.

	Tetiles of glycemic load				Tertiles of total dietary fiber			
	T1(n = 100) < 123.9	T2(n = 100) 123.9–172.7	T3(n = 100) > 172.7	P^2^	T1(n = 100) < 13.5	T2(n = 100) 13.5–19.6	T3(n = 100) > 19.6	P^a^
Foot ulcer length	1.22 ± 0.16	1.23 ± 0.13	1.28 ± 0.16	0.96	1.46 ± 0.14	1.10 ± 0.12	1.17 ± 0.13	0.16
Foot ulcer width	0.76 ± 0.09	0.68 ± 0.07	0.82 ± 0.09	0.47	0.82 ± 0.07	0.67 ± 0.07	0.79 ± 0.07	0.34
Foot ulcer depth	0.35 ± 0.06	0.21 ± 0.05	0.28 ± 0.06	0.25	0.32 ± 0.06	0.24 ± 0.05	0.28 ± 0.05	0.60

All values are means ± standard error (SE); all values are adjusted for age, gender and energy intake, smoking, socioeconomic status, physical activity, insulin intake, more than 2years of diabetes, debridement, good shoe condition, good socks condition, diabetic foot ulcer history, body mass index.

^a^Obtained from ANCOVA.

Among all the carbohydrate quality indices and quantity of carbohydrate intake examined “Tables 6–8”, the results revealed that only the whole grain to total grain ratio significantly influenced the length of the diabetic foot ulcer. Comparing participants with the lowest consumption of whole grain to those with the highest intake, the former group had a larger length of the ulcer. This relationship remained significant even after adjusting for potential confounders such as age, gender, total energy intake, smoking, physical activity, good shoe condition, HbA1c, neuropathy, peripheral arterial disease (PAD), infection and BMI “Table 8”.

**Table 6:** Multivariable-adjusted odds ratio of high diabetic foot ulcer indices based on tertiles of carbohydrate intake.

	Tetiles of carbohydrate intake			
	T1 (n = 100)	T2 (n = 100)	T3 (n = 100)	P-trend
Foot ulcer length				
Crude	1	0.95(0.53,1.70)	1.01(0.56,1.77)	1.00
Model 1	1	0.72(0.37,1.40)	0.59(0.24,1.43)	0.244
Model 2	1	0.61(0.29,1.27)	0.49(0.18,1.30)	0.148
Model 3	1	0.69(0.32,1.48)	0.61(0.22,1.64)	0.303
Foot ulcer width				
Crude	1	1.01(0.55,1.79)	1.19(0.66,2.12)	0.554
Model 1	1	0.82(0.42,1.61)	0.86(0.36,2.08)	0.745
Model 2	1	0.74(0.35,1.57)	0.74(0.27,1.99)	0.575
Model 3	1	0.89(0.40,1.95)	0.97(0.35,2.66)	0.920
Foot ulcer depth				
Crude	1	0.82(0.45,1.51)	0.41(0.21,0.81)	0.111
Model 1	1	0.80(0.39,1.65)	0.46(0.16,1.28)	0.148
Model 2	1	0.67(0.29,1.52)	0.36(0.11,1.14)	0.080
Model 3	1	0.74(0.31,1.76)	0.44(0.13,1.41)	0.140
Wagner grade				
Crude	1	1.05(0.51,2,22)	0.86(0.40,1.83)	0.704
Model 1	1	0.96(0.41,2.21)	0.74(0.23,2.33)	0.619
Model 2	1	0.89(0.34,2.32)	0.45(0.12,1.72)	0.245
Model 3	1	0.85(0.31,2.36)	0.42(0.10,1.69)	0.212

All values are odds ratios and 95% confidence intervals. Model 1: Adjusted for age, gender, energy intake. Model 2: Further adjustment for smoking, socioeconomic status, physical activity, history of diabetic foot ulcer, history of diabetes for more than two years, insulin consumption, condition of shoes and socks, debridement, neuropathy, PAD, infection, socks condition, metformin, offloading, HbA1c. Model 3: Further adjustment for BMI.

**Table 8:** Multivariable-adjusted odds ratio of high diabetic foot ulcer indices based on tertiles of whole grain to total grain and carbohydrate of solid food to total carbohydrate.

	whole grain/total grain				carbohydrate of solid food/ total carbohydrate			
	T1(n = 101) < 0.14	T2(n = 98) 0.14–0.40	T3(n = 101) > 0.40	P-trend	T1(n = 110) < 0.86	T2(n = 89) 0.86–0.91	T3(n = 101) > 0.91	P-trend
Foot ulcer length								
Crude	1	0.85(0.48,1.50)	0.54(0.30,0.97)	0.04	1	1.46(0.81,2.61)	1.18(0.67,2.09)	0.53
Model1	1	0.85(0.48,1.53)	0.56(0.31,1.03)	0.03	1	1.42(0.78,2.56)	1.20(0.67,2.14)	0.51
Model2	1	0.81(0.43,1.52)	0.55(0.29,1.04)	0.03	1	1.57(0.83,2.96)	1.38(0.74,2.58)	0.52
Model3	1	0.71(0.37,1.37)	0.52(0.27,1.00)	0.03	1	1.37(0.71,2.65)	1.29(0.68,2.46)	0.63
Foot ulcer width								
Crude	1	0.84(0.47,1.50)	0.67(0.37,1.20)	0.18	1	1.13(0.63,2.01)	0.77(0.43,1.37)	0.40
Model1	1	0.85(0.47,1.54)	0.71(0.39,1.30)	0.27	1	1.09(0.60,1.96)	0.77(0.43,1.39)	0.40
Model2	1	0.81(0.42,1.55)	0.72(0.38,1.37)	0.26	1	1.01(0.53,1.93)	0.82(0.43,1.55)	0.49
Model3	1	0.67(0.34,1.32)	0.70(0.36,1.36)	0.27	1	0.92(0.47,1.80)	0.75(0.39,1.46)	0.40
Foot ulcer depth								
Crude	1	0.89(0.47,1.67)	0.90(0.48,1.68)	0.74	1	1.23(0.67,2.26)	0.59(0.30,1.13)	0.13
Model1	1	0.97(0.50,1.86)	1.01(0.52,1.93)	0.98	1	1.27(0.68,2.37)	0.63(0.32,1.23)	0.21
Model2	1	1.23(0.59,2.59)	1.29(0.61,2.68)	0.54	1	1.84(0.87,3.90)	1.64(0.76,3.53)	0.12
Model3	1	1.17(0.54,2.57)	1.45(0.67,3.12)	0.41	1	2.16(0.99,4.74)	1.60(0.71,3.61)	0.07
Wagner grade								
Crude	1	1.10(0.54,2.26)	0.68(0.31,1.47)	0.34	1	1.29(0.62,2.76)	0.82(0.38,1.75)	0.64
Model1	1	1.16(0.56,2.41)	0.74(0.33,1.64)	0.48	1	1.27(0.60,2.65)	0.84(0.39,1.82)	0.70
Model2	1	1.37(0.60,3.11)	0.88(0.36,2.12)	0.77	1	1.10(0.47,2.52)	1.01(0.42,2.38)	0.94
Model3	1	1.17(0.50,2.78)	0.73(0.29,1.83)	0.51	1	1.02(0.42,2.47)	0.93(0.37,2.32)	0.90

All values are odds ratios and 95% confidence intervals. Model 1: Adjusted for age, gender, energy intake. Model 2: Further adjustment for smoking, socioeconomic status, physical activity, history of diabetic foot ulcer, history of diabetes for more than two years, insulin consumption, condition of shoes and socks, debridement, neuropathy, PAD, infection, socks condition, metformin, offloading, HbA1c. Model 3: Further adjustment for BMI.

**Table 7:** Multivariable-adjusted odds ratio of high diabetic foot ulcer indices based on tertiles of glycemic load and dietary fiber.

	Tetiles of glycemic load				Tertiles of total dietary fiber			
	T1(n = 100) < 123.9	T2(n = 100) 123.9–172.7	T3(n = 100) > 172.7	P-trend	T1(n = 100) < 13.5	T2(n = 100) 13.5–19.6	T3(n = 100) > 19.6	P-trend
Foot ulcer length								
Crude	1	1.47(0.82,2.61)	1.09(0.60,1.96)	0.76	1	0.70(0.39,1.26)	0.80(0.45,1.43)	0.46
Model1	1	1.21(0.63,2.32)	0.79(0.32,1.91)	0.65	1	0.68(0.37,1.27)	0.69(0.36,1.33)	0.28
Model2	1	1.19(0.58,2.45)	0.77(0.29,2.05)	0.64	1	0.60(0.30,1.17)	0.56(0.27,1.15)	0.20
Model3	1	1.29(0.61,2.71)	0.86(0.31,2.35)	0.83	1	0.59(0.29,1.19)	0.59(0.28,1.22)	0.28
Foot ulcer width								
Crude	1	1.19(0.66,2.14)	1.30(0.72,2.33)	0.37	1	0.76(0.42,1.37)	1.04(0.58,1.85)	0.88
Model1	1	1.08(0.65,2.09)	1.20(0.50,2.90)	0.67	1	0.77(0.41,1.46)	0.96(0.50,1.84)	0.93
Model2	1	1.07 0.51,2.24)	1.13(0.42,3.01)	0.78	1	0.80(0.40,1.59)	0.88(0.43,1.81)	0.90
Model3	1	1.26(0.58,2.73)	1.41(0.51,3.88)	0.54	1	0.78(0.38,1.59)	0.88(0.42,1.85)	0.92
Foot ulcer depth								
Crude	1	0.41(0.22,0.77)	0.36(0.19,0.69)	0.12	1	0.74(0.39,1.37)	0.66(0.35,1.24)	0.20
Model1	1	0.38(0.18,0.79)	0.37(0.13,0.99)	0.23	1	1.05(0.53,2.08)	0.99(0.48,2.02)	0.98
Model2	1	0.35(0.15,8.81)	0.34(0.11,1.04)	0.32	1	1.23(0.57,2.66)	0.86(0.38,1.97)	0.88
Model3	1	0.40(0.17,0.95)	0.42(0.13,1.32)	0.27	1	1.42(0.62,3.22)	1.01(0.42,2.37)	0.85
Wagner grade								
Crude	1	0.56(0.26,1.19)	0.71(0.34,1.47)	0.34	1	1.31(0.63,2.71)	0.85(0.39,1.86)	0.70
Model1	1	0.47(0.20,1.10)	0.56(0.18,1.70)	0.25	1	1.53(0.70,3.37)	0.95(0.39,2.27)	0.87
Model2	1	0.37(0.14,1.01)	0.34(0.09,1.24)	0.55	1	2.31(0.93,5.71)	1.01(0.37,2.65)	0.85
Model3	1	0.34(0.12,0.95)	0.32(0.08,1.22)	0.53	1	2.04(0.79,5.25)	0.84(0.30,2.32)	0.95

All values are odds ratios and 95% confidence intervals. Model 1: Adjusted for age, gender, energy intake. Model 2: Further adjustment for smoking, socioeconomic status, physical activity, history of diabetic foot ulcer, history of diabetes for more than two years, insulin consumption, condition of shoes and socks, debridement, neuropathy, PAD, infection, socks condition, metformin, offloading, HbA1c. Model 3: Further adjustment for BMI.

## Discussion

We found that a higher ratio of whole grain to total grain intake was associated with a smaller diabetic foot ulcer length. The findings suggest that individuals of older age may tend to consume lower amounts of carbohydrates. Furthermore, a significant association was observed between gender and carbohydrate intake, with a higher proportion of men consuming greater amounts compared to women. This finding supports the notion that gender can be a potential risk factor for diabetic ulcers and poor glycemic control in men, which is consistent with previous research [54–56]. Additionally, a higher proportion of smokers were found in the third tertile (T3) of carbohydrate intake, indicating a potential relationship between smoking habits and higher carbohydrate consumption. This finding supports previous studies that have identified smoking as a risk factor for diabetic foot ulcers [57–59]. In addition, a meta-analysis revealed that smokers are likely to have lower levels of circulating vitamin D than non-smokers [60]. Besides, it was indicated in another meta-analysis that severe vitamin D deficiency is significantly related to an increased risk of DFU [61]. Another way to explain this is that insulin resistance can be influenced not only by excessive carbohydrate intake, particularly from low-quality sources, but also by smoking status, with both current and former smokers exhibiting higher levels of insulin resistance compared to non-smokers. [62–64]. Furthermore; individuals with greater carbohydrate consumption tend to have a higher overall energy intake and an increased intake of essential nutrients. However, we did not identify a significant relationship between glycemic load, fiber, and the quantity of carbohydrate consumed with the size, depth or Wagner grade of the diabetic foot ulcer. Numerous studies have explored the impact of nutrition on diabetes outcomes in adults [65–71]. However, this finding contrasts with previous studies that have shown a positive association between glycemic control and factors relevant to the management of diabetic foot ulcers [19,22,72]. There are several potential reasons why we did not observe these associations in our study. Firstly, variations in study design, sample characteristics, and methodologies for assessing dietary factors may contribute to the observed discrepancies. Secondly, the duration of diabetes and overall glycemic control of the participants may have influenced the results. It is possible that participants with better glycemic control or shorter durations of diabetes had smaller or less severe diabetic foot ulcers. This could have weakened the associations between the foot ulcers and factors like glycemic load, fiber intake, and total carbohydrate consumption. Additionally, the development of diabetic foot ulcers is a complex process involving multiple factors beyond glycemic control and dietary factors alone. Factors such as vascular disease, neuropathy, and foot care practices also play significant roles in the development and progression of diabetic foot ulcers [73–76]. The interplay of these factors might have overshadowed the direct associations we hypothesized. Our finding indicates that a higher ratio of whole grains to total grains is associated with smaller diabetic foot ulcers. This highlights the potential benefits of incorporating more whole grains into the diet of individuals with diabetic foot ulcers. Whole grains may possess protective properties that contribute to improved wound healing. Whole grains are rich in beneficial nutrients, including vitamins, minerals, antioxidants, and phytochemicals. These nutrients work synergistically to support optimal metabolic functioning and may help improve insulin sensitivity, which is essential for maintaining blood sugar levels within a healthy range [77,78]. In addition, whole grains are a source of prebiotics that promote the growth of beneficial gut bacteria. A healthy gut microbiota has been associated with improved glucose metabolism and better glycemic control [79–82]. Moreover, whole grains tend to be more filling and satisfying compared to refined grains. The increased satiety provided by whole grains can help regulate appetite, prevent overeating, and support weight management [83]. Gut microbes have the ability to generate metabolites like short-chain fatty acids (SCFAs) through the fermentation of fiber and other phenolic compounds found in whole grains (WGs) [84]. These SCFAs have been demonstrated to stimulate the production of hormones related to appetite, such as glucagon-like peptide-1 and peptide YY [85–87]. Plus, whole grains, which contain high levels of viscous and fermentable fiber like β-glucan, have been proven to delay gastric emptying and extend the release of cholecystokinin when consumed with a meal containing fat [84,88,89]. This effect potentially contributes to an increased sense of fullness or satiety. Maintaining a healthy weight is crucial for glycemic control, as excess body weight is associated with insulin resistance and elevated blood sugar levels [90].

### Strengths and limitations

Our study is the first to comprehensively evaluate both the quality and quantity of carbohydrate intake in patients with diabetes suffering from diabetic foot ulcers. We considered different indices for the quality of carbohydrates, such as glycemic load, fiber intake, and the whole grain to total grain ratio. Additionally, adjusting for potential confounders such as age, gender, energy intake, smoking, physical activity, shoe condition, neuropathy, PAD, HbA1c, and BMI strengthens the validity of the observed associations.

However, several limitations should be acknowledged. Firstly, the cross-sectional design of the study limits the ability to establish causal relationships between carbohydrate intake and diabetic foot ulcer characteristics. Longitudinal studies are needed to further elucidate the temporal associations and potential mechanisms involved. Secondly, we evaluated dietary intakes using 24-hour dietary recalls. However, this method is prone to recall bias, affecting both the identification of consumed foods and the quantification of portion sizes. To mitigate this type of error, we gathered the dietary data through the use of highly-trained interviewers. Additionally, to reduce this error, we used three 24-hour dietary recalls, which according to the study published in 2016, three 24-hour dietary recalls can accurately estimate usual intake [91]. While we attempted to control the results for the main diabetes-related medications such as insulin, and metformin, as well as for wound care interventions like debridement, shoe and sock status, offloading, we did not control for all medications. However, in a trial the authors acknowledged that some patients were receiving antibiotic treatment, without any signs of infection. The use of antibiotics may have introduced a potential confounding factor. But their analysis indicated it did not significantly impact the study’s outcomes. Specifically, the multivariate analysis did not find a significant association between antibiotic treatments and wound healing. Furthermore, a subgroup analysis revealed no substantial differences in healing rates or duration between patients who were taking antibiotics at the start of the study and those who were not [92].

Lastly, the study did not explore the potential influence of inflammation and comorbidities on the observed associations, which should be considered in future investigations.

## Conclusion

In conclusion, this study provides valuable insights into the associations between carbohydrate intake and diabetic foot ulcer characteristics among individuals with type 2 diabetes. The findings highlight the importance of considering both the quantity and quality of carbohydrate intake, specifically increasing whole grain intake in relation to diabetic foot ulcer characteristics. Further research is warranted to confirm these findings and investigate the underlying mechanisms. This could ultimately contribute to the development of targeted dietary interventions for individuals with diabetic foot ulcers in the context of type 2 diabetes.

## Supporting information

S1 Dataset Supporting information. (XLSX)
